# Quantitative imaging of tumour blood flow by contrast-enhanced magnetic resonance imaging

**DOI:** 10.1054/bjoc.2001.2157

**Published:** 2001-11

**Authors:** S Pahernik, J Griebel, A Botzlar, T Gneiting, M Brandl, M Dellian, A E Goetz

**Affiliations:** Institute for Surgical Research, Departments of Otorhinolaryngology, Anesthesiology, Klinikum Grosshadern, University of Munich, Marchioninistrasse 15, Munich, 81377, Germany; Institute for Radiation Biology, National GSF Research Center for Environment and Health, Neuherberg, Germany

**Keywords:** tumour, blood flow, MRI, methodology

## Abstract

Tumour blood flow plays a key role in tumour growth, formation of metastasis, and detection and treatment of malignant tumours. Recent investigations provided increasing evidence that quantitative analysis of tumour blood flow is an indispensable prerequisite for developing novel treatment strategies and individualizing cancer therapy. Currently, however, methods for noninvasive, quantitative and high spatial resolution imaging of tumour blood flow are rare. We apply here a novel approach combining a recently established ultrafast MRI technique, that is T _1_-relaxation time mapping, with a tracer kinetic model. For validation of this approach, we compared the results obtained in vivo with data provided by iodoantipyrine autoradiography as a reference technique for the measurement of tumour blood flow at a high resolution in an experimental tumour model. The MRI protocol allowed quantitative mapping of tumour blood flow at spatial resolution of 250 × 250 μm^2^. Correlation of data from the MRI method with the iodantipyrine autoradiography revealed Spearman's correlation coefficients of Rs = 0.851 (r = 0.775, *P* < 0.0001) and Rs = 0.821 (r = 0.72, *P* = 0.014) for local and global tumour blood flow, respectively. The presented approach enables noninvasive, repeated and quantitative assessment of microvascular perfusion at high spatial resolution encompassing the entire tumour. Knowledge about the specific vascular microenvironment of tumours will form the basis for selective antivascular cancer treatment in the future. © 2001 Cancer Research Campaign http://www.bjcancer.com


British Journal of Cancer (2001) 85(11), 1655–1663
© 2001 Cancer Research Campaign
doi: 10.1054/ bjoc.2001.2157, available online at http://www.idealibrary.com on IDEAL

### Quantitative imaging of tumour blood flow by contrast-enhanced magnetic resonance imaging

**S Pahernik[1], J Griebel[2], A Botzlar[1], T Gneiting[2], M Brandl[2], M Dellian[1,3] and AE Goetz[4]**

[1]Institute for Surgical Research, Departments of [3]Otorhinolaryngology and [4]Anesthesiology, Klinikum Grosshadern, University of Munich, Marchioninistrasse 15, 81377 Munich, Germany; [2]Institute for Radiation Biology, National GSF Research Center for Environment and Health, Neuherberg, Germany

**Summary** Tumour blood flow plays a key role in tumour growth, formation of metastasis, and detection and treatment of malignant tumours. Recent investigations provided increasing evidence that quantitative analysis of tumour blood flow is an indispensable prerequisite for developing novel treatment strategies and individualizing cancer therapy. Currently, however, methods for noninvasive, quantitative and high spatial resolution imaging of tumour blood flow are rare. We apply here a novel approach combining a recently established ultrafast MRI technique, that is $T_1$-relaxation time mapping, with a tracer kinetic model. For validation of this approach, we compared the results obtained in vivo with data provided by iodoantipyrine autoradiography as a reference technique for the measurement of tumour blood flow at a high resolution in an experimental tumour model. The MRI protocol allowed quantitative mapping of tumour blood flow at spatial resolution of $250 \times 250\ \mu m^2$. Correlation of data from the MRI method with the iodantipyrine autoradiography revealed Spearman's correlation coefficients of Rs = 0.851 (r = 0.775, $P < 0.0001$) and Rs = 0.821 (r = 0.72, $P = 0.014$) for local and global tumour blood flow, respectively. The presented approach enables noninvasive, repeated and quantitative assessment of microvascular perfusion at high spatial resolution encompassing the entire tumour. Knowledge about the specific vascular microenvironment of tumours will form the basis for selective antivascular cancer treatment in the future. © 2001 Cancer Research Campaign http://www.bjcancer.com

**Keywords**: tumour; blood flow; MRI; methodology

Tumour blood flow as a result of angiogenesis plays a key role in growth, formation of metastasis, as well as detection and treatment of malignant solid tumours (Folkman, 1995). Quantitative analysis of tumour blood flow is of paramount importance for the assessment of microvascular function, for the successful delivery of therapeutic agents, for improving diagnostic procedures and current treatment options, and for the development of novel antivascular treatment strategies (Jain, 1998). Knowledge about tumour vascular pathophysiology is highly relevant for the prognosis of cancer patients (Weidner et al, 1991; Hoeckel et al, 1996) and can be used to plan and to select optimal treatment regimes for patients, thus individualizing cancer therapy.

Sensitivity of solid tumours to various treatment modalities is critically determined by tumour blood flow. Blood flow determines microenvironmental parameters such as tissue oxygenation, pH distribution, bioenergetic status and the nutrient supply which influence sensitivity of malignant cells to anticancer treatment procedures (Vaupel et al, 1989). Cells outgrowing their nutrient supply induce hypoxic regions within the tumour with quiescent and necrotic cells. Hypoxic cells are more resistant to most treatment modalities due to their altered physiologic and metabolic state. With respect to radiotherapy, treatment is greatly dependent upon oxygen supply to the tumour which is governed by the blood flow. In chemo- and immunotherapy, sufficient vascular supply and blood flow is necessary for the delivery of cytotoxic agents or cells to the tumour. In the case of hyperthermia, tumour blood flow is the main route for heat transfer leading to a reduced efficacy in highly perfused areas of the tumour.

A noninvasive in vivo approach enabling quantitative imaging of tumour blood flow is highly desirable and clinically powerful, in particular if the method would encompass the entire tumour and could be repeated frequently. Magnetic resonance imaging (MRI) is a versatile technique which offers the potential to fulfil these requirements (Henderson et al, 2000). Recent technical improvements concerning ultrafast MRI protocols such as echo planar imaging or snapshot FLASH which allow more rapid acquisition times and/or higher spatial resolution widen the potential for functional applications. Several studies have shown that differences in tumour vasculature are associated with different patterns of contrast enhancement (Mayr et al, 1996; Degani et al, 1997; Hawighorst et al, 1998). However, an understanding of local tumour pathophysiology, and thus, of signal enhancement patterns in MRI after the administration of paramagnetic contrast agents, is required in order to attribute parameters obtained by MRI to morphology and function of tumour vasculature. Recently, a ultrafast MRI technique, that is T1 mapping ensuring linearity between tracer concentration and MR signal, has been introduced to obtain concentration time data from MR images (Deichmann and Haase, 1992). This technique was used to assess microcirculatory changes via a perfusion index in patients with rectal carcinoma during chemoirradiation (de Vries et al, 2000). Moreover, the authors could demonstrate that this perfusion index, as measured before onset of treatment, is predictive for therapy outcome (de Vries et al, 2001). However, the perfusion index is influenced not only by tumour perfusion, but also by capillary permeability-surface product. In order to improve characterization of tumour microcirculation

*Received 20 February 2001*
*Revised 12 July 2001*
*Accepted 30 July 2001*

*Correspondence to:* S Pahernik, Department of Urology, Johannes-Gutenberg University Mainz, Langenbeckstrasse 1, 550101 Mainz tracer kinetic approaches are necessary allowing reliable quantification of tumour blood flow.

The aim of the present study was to validate a promising approach combining the ultrafast $T_1$ mapping technique (de Vries et al, 2000) with a more complex tracer kinetic model (Griebel et al, 1997) which takes into account the specific pharmacokinetic properties of the widely used contrast agent gadopentetate dimeglumine (Gd-DTPA). The approach has been validated in vivo by correlating the results with data obtained from iodoantipyrine autoradiography as the reference method for the measurement of tumour blood flow at high spatial resolution.

### MATERIAL AND METHODS

### Dynamic MR imaging

MRI measurements were performed on a 400 MHz magnetic resonance system with 9.4 T (DMX 400, Bruker, Karlsruhe, Germany). For calculation of $T_1$ maps, an inversion recovery snapshot FLASH pulse sequence was implemented into the MR system (Nekolla et al, 1992). After one initial non-selective fast passage adiabatic inversion pulse, 20 rapid $T_1$-weighted snapshot FLASH images were acquired within 2.5 s. To guarantee a sufficiently accurate sampling of fast relaxation dynamics in contrast-enhanced tissue and blood, a short repetition time and gradient echo time (TR = 2.3 ms, TE = 1.1 ms, flip angle = 5˚) was used. Data were acquired with a matrix size of 64 × 128 and zero filled to a 128 × 128 matrix. A field of view of 25–30 mm provided an in-plane resolution of 250 × 250 $\mu m^2$. The slice thickness was 750 µm. After bolus administration of a paramagnetic contrast agent, very short $T_1$ relaxation times occur in tissue, and especially in arterial blood. The accurate evaluation of short $T_1$ relaxation times depends essentially on the fast acquisition of the early relaxation dynamics. Since the image signal intensity of a snapshot FLASH image mostly represents the longitudinal magnetization, which is present at the beginning of the acquisition of the zero-phase encoding line, a phase-encoding scheme sampling the low frequency part of raw matrix at the beginning of each sequence improves the measurement of small $T_1$ values as compared to conventional sequential phase encoding. Therefore, our measurements employed an asymmetrical sequential phase-encoding scheme, where the initial 16 (high frequency) rows of raw data matrix are cut off and appended at the end of k-space sampling.

From the acquired $T_1$-weighted Snapshot FLASH images, $T_1$ maps are calculated using a 3-parameter least squares fit on a pixel-by-pixel basis taking into account both $T_1$ relaxation and progressive saturation effects (Nekolla et al, 1992). To ensure that $B_1$ inhomogeneities do not degrade the accuracy of the $T_1$ estimates a low flip angle approximation was used (Deichmann and Haase, 1992). This approximation provides stable $T_1$ estimates for flip angles between 2˚ to 8˚. This methodological approach ensures linearity between concentration of the contrast agent and $T_1$ relaxation rate (Donahue et al, 1994). This approach has been recommended for dynamic MR studies whenever it is planned to perform a quantitative analysis by tracer kinetic modelling (Diesbourg et al, 1992; Tong et al, 1993; Donahue et al, 1994).

### Concentration time data

The contrast agent gadopentetate dimeglumine (Gd-DTPA, Magnevist™, Schering AG, Berlin, Germany) was used in this study. Gadopentetate dimeglumine is a low molecular-weight tracer (MW 548 d) with extracellular distribution and renal excretion which behaves in tumours as intermediate between a freely diffusible and an intravascular tracer.

For the dynamic studies, 4 transaxial precontrast $T_1$ maps, which were positioned to intersect both heart and tumour, were acquired. Next, 0.05 mmol $kg^{-1}$ body weight gadopentetate dimeglumine was infused at 0.125 ml $min^{-1}$ for 4 min. A total of 47 $T_1$ maps were acquired within 60 min after onset of contrast agent infusion. To guarantee accurate $T_1$ maps during continuous sequence repetition a full recovery of the longitudinal magnetization between the application of subsequent inversion pulses is necessary. Therefore, temporal resolution of $T_1$ mapping is limited to 10s during the initial sampling period of 2 min, which ensures full recovery of $T_1$ magnetization. As a consequence, a prolonged bolus of 4 min infusion time of Gd-DTPA was used to sample sufficient data points for the precise assessment of the up-slope part of concentration–time curves in arterial blood and tissue.

To measure the arterial input function, regions of interest (ROIs) were defined in the centre of the cavum of the left ventricle. To exclude motion-derived artifacts during measurements of gadopentetate dimeglumine concentration within the left ventricle, only time courses from pixels with a steep initial slope of $T_1$ relaxation rate data (above the 75% quartile) were included for calculation of the arterial input function (Rempp et al, 1994). A flow compensation was not necessary. The evaluation of the arterial input function by using inversion recovery snapshot FLASH sequences has been validated previously by an independent study applying sequential arterial and venous blood sampling during MRI (Fritz-Hansen et al, 1996).

Concentration time curves, C(t), for arterial blood and tumour tissue were calculated from changes in $T_1$ relaxation rates [ΔR(t)] between the pre- and postcontrast images according to the following formula:

$$\Delta R_1(t) = \frac{1}{T_{1\,post}(t)} - \frac{1}{T_{1\,per}} = \beta C \qquad (1)$$

whereas $T_{1pre}$ and $T_{1post}(t)$ denote the $T_1$ relaxation time before and after the onset of gadopentetate dimeglumine injection, respectively, and $\beta$ denotes the relaxivity of the contrast agent. It was assumed that $\beta$ is identical for blood and tissue which has previously been shown experimentally (Donahue et al, 1994).

The basic assumption of Eq. (1) is the linearity between $T_1$ relaxation rate and the gadopentetate dimeglumine concentration. To verify this assumption and to estimate a reliable in vivo value for the relaxivity, β, an additional study using the same experimental model was performed. Hereby, gadopentetate dimeglumine, at doses of 0.05, 0.1, 0.15 and 0.3 mmol $kg^{-1}$ body weight, was infused via the superior vena cava for 4 min at an infusion rate of 0.125 ml $min^{-1}$. 30 min afterwards, $T_1$ maps were acquired with the aforementioned methodology, and blood samples were taken simultaneously from the carotid artery via a polyethylene catheter (Portex Ltd, Hythe, Kent, England) which was inserted before onset of the MR experiments. Immediately afterwards, blood was centrifuged, and $T_1$ relaxation rate was measured ex vivo in the diluted plasma aliquots. Next, the plasma was frozen until analysis of the concentration of gadopentetate dimeglumine by means of inductive coupled plasma atomic spectroscopy.

*© 2001 Cancer Research Campaign*

### Tracer kinetic model for quantification of tumour blood flow

For the quantification of tumour blood flow we applied a novel tracer kinetic model (Griebel et al, 1997). The model is an extension of an approach originally developed by Meier and Zierler (1963). Although the approach of Meier and Zierler was designed originally for intravascular tracers, their assumptions are not restricted to this use. It is in particular suitable for extracellular contrast agents, and for an arbitrarily shaped bolus at the arterial entry into the tissue, and is consequently not restricted to first pass studies. The basic assumptions for the tracer kinetic model are: (a) stationarity of flow distribution with respect to the observed time scale; (b) representativity of the tracer for total fluid; and (c) inertness of the tracer showing no interactions with tissue and proteins.

The concentration–time data were fitted to following mathematical expression using a nonlinear algorithm:

$$C_t(t) = p_t\left[\int_0^t C_a(\tau)d\tau - \left(\int_0^t C_a(\tau)d\tau\right) \otimes h(t)\right] \tag{2}$$

where $C_t(t)$ and $C_a(t)$ represent concentration–time curves in tumour tissue and arterial blood, respectively, $p_t$ denotes tissue perfusion of the tumour and *h(t)* transit time distribution, which is the distribution of transit times of tracer molecules through the tissue after an instantaneous bolus.

For an extracellular tracer such as gadopentetate dimeglumine, the capillary permeability is so low that only a fraction $\alpha$ of the tracer enters the interstitial space, while the remaining fraction (1-$\alpha$), behaves exactly like an intravascular tracer. In line with Zierler (1963), the frequency function h(t) for extracellular tracers such as gadopentetate dimeglumine can be split into two parts: the fraction $\alpha$ of tracer molecules entering the interstitial space and the fraction 1-$\alpha$ remaining in the vascular space:

$$h(t) = (1-\alpha)h_{iv}(t) + \alpha h_{ec}(t) \tag{3}$$

where $h_{iv}(t)$ is the frequency function of transit times of the intravascular tracer molecules, and $h_{ec}(t)$ is the frequency function of transit times of the extracellular tracer molecules.

Let $h_{in}(t)$ be the interstitial frequency function of transit times from capillary through interstitial space back to capillary, then $h_{ec}(t)$ is the convolution of $h_{iv}(t)$ and $h_{in}(t)$:

$$h_{ec}(t) = h_{iv}(t) \otimes h_{in}(t) \tag{4}$$

As a first approach, the functions $h_{iv}(t)$ and $h_{in}(t)$ were parameterized by monoexponential approaches (Griebel et al, 2001):

$$\text{Intravascular frequency function:}\quad h_{iv}(t) = I_b e^{-I_b t}$$
$$\text{Interstitial frequency function:}\quad h_{in}(t) = I_d e^{-I_d t} \tag{5}$$

Furthermore, assuming a monoexponential increase and thereafter a 3-exponential decay of the arterial input function, $C_a(t)$, the concentration–time curve, $C_t(t)$, in Eq. (2) can be expressed analytically. Under the assumption, that the relaxivity, $\beta$, is identical for blood and tissue, $\beta$ can be eliminated in Eq. (2) and calculations can be performed on the base of relaxation rate changes, $\Delta R(t)$, as described in Eq. (1). As a consequence, tumour blood flow $p_t$, the extraction fraction $\alpha$, and $I_b$ and $I_d$ can be quantified by least squares fitting from Eq. [2]. $1/I_b$ represents the mean transit time of tracer molecules through the intervascular space (MTT) iv and $1/I_d$ represents the mean transit time of tracer molecules through the interstitial space (MTT) in.

The fit parameters were corrected for differences of haematocrit in large vessels and capillaries. Haematocrit in large vessels was measured at the end of MRI experiments and found to be 0.44 ± 0.02, while microvascular haematocrit was assumed to be 0.18 which was quantified in the same tumour model in a previous study (Goetz, 1987).

### In vivo MR measurements of tumour blood flow

Following approval of the local ethics committee, experiments were performed on male Syrian golden hamsters (50–60 g body weight) in accordance with the UKCCCR ‘Guidelines for the welfare of animals in experimental neoplasia’ (Workman et al, 1998). For all surgical procedures, animals were anesthetized intraperitoneally with 60 mg $kg^{-1}$ body weight pentobarbital (Nembutal, Sanofi-Leva, Hannover, Germany). Cells (5 × $10^6$ cells) of the amelanotic hamster melanoma A-Mel-3 (Fortner et al, 1961) were implanted subcutaneously at the right flank near the upper foreleg at the level of the heart. 3 days after implantation when tumour had reached palpable size, a pin was implanted into the tumour to facilitate orientation of perfusion maps obtained by MRI and autoradiography. 5 days after implantation, when tumours had reached a volume of 90–140 $mm^3$, polyethylene catheters (Portex Ltd, Hythe, Kent, England) were inserted into the right carotid artery for arterial blood pressure measurements, into the superior vena cava for infusion of the tracer, and into the femoral artery for arterial blood sampling. A specially designed animal holder for the MRI system allowed inhalation anaesthesia, measurement of mean arterial blood pressure, and control of body temperature. Animals were anesthetized with 1.5% isoflurane. The temperature in the animal holder was kept constant at 28˚C. Only animals with a stable mean arterial blood pressure between 60 and 80 mmHg during experiments were enrolled into this study in order to exclude blood pressure mediated alterations of tumour blood flow during experiments. 7 animals have been included in the study.

### Autoradiographic measurements of tumour blood flow

Immediately after MRI investigations the hamster was removed from the MRI system for tumour perfusion autoradiography. Tumour blood flow was quantified by the autoradiographic tissue equilibration technique modified for the A-Mel-3 tumour as described (Kuhnle et al, 1992). The method is based on the tissue uptake of the inert and freely diffusible compound [4-N-methyl-$^{14}C$] iodoantipyrine (MW 314, IAP) and relies on the well-established tracerkinetic model of Kety for freely diffusible tracers (Kety, 1960). This tracer (Du Pont-NEN; 40 µCi dissolved in 0.55 ml 0.9% NaCl) was administered intravenously for 30 s while arterial blood samples were collected every 5 s for determination of blood concentration. Immediately after the end of infusion, tumour tissue was rapidly resected and immediately frozen in liquid nitrogen. The activity of $^{14}C$ in arterial blood samples was measured using a liquid scintillation counter (Rack Beta 1219, LKB Wallac, Turku, Finland).

Tumours were cut into serial cryosections consecutive for tumour blood flow autoradiography (20 µm thickness) and for

*© 2001 Cancer Research Campaign*

histology (5 µm thickness). Tumours could be identified in histological sections which were stained with haematoxylin and eosin. Thus, regions of interest could be defined. Sections for autoradiography were dried and exposed to X-ray film (NMC, Eastman Kodak, Rochester, NY) for 2 weeks together with calibrated $^{14}C$ tissue standards (Amersham Buchler GmbH, Braunschweig, Germany). Images of the autoradiography showing $^{14}C$ tissue distribution were acquired by a CCD-video camera (XC-77, Sony, Cologne, Germany) which was coupled to a macroviewer and digitized (IBAS 2.0, Kontron, Eching, Germany). Local tumour blood flow was calculated from grey levels according to Sakurada et al (1978) using the equation given by Kety (1960).

### Comparison of tumour blood flow images acquired by MRI and by autoradiography

For correlation of perfusion data by MRI and autoradiography, the MRI images (125 × 125 pixels) were transferred to 512 × 512 pixels with 255 grey levels by linear interpolation. 4 autoradiographic specimens (20 µm) lying in the level of the MRI map (750 µm) were averaged to obtain a corresponding image of the spatial heterogeneity of tumour blood flow. Perfusion maps of MRI and autoradiography were adjusted by a specially designed image transforming system (Kuhnle et al, 1992) (IBAS 2.0, Kontron, Eching, Germany) to allow quantification of tumour blood flow in corresponding areas of MRI and autoradiography images by digital image analysis.

### Statistics

Data are expressed as mean ± standard error of the mean. Relation between blood flow data obtained by MRI and autoradiography was analysed by the Spearman correlation coefficient $R_s$ and linear regression. A $P$ value less than 0.05 was regarded as statistically significant. Agreement between the 2 methods for the quantification of tumour blood flow was evaluated and calculated in terms of bias and precision as described by Bland and Altman (1986).

### RESULTS

The MRI protocol used allowed dynamic $T_1$ mapping with high spatial resolution (in-plane resolution: 250 × 250 µm$^2$, slice thickness: 750 µm) with an acquisition time of 2.5 s. Maps of $T_1$ relaxation times in a transaxial section of the animal at different times before and after injection of gadopentetate dimeglumine are shown in Figure 1. From these images, the concentration of gadopentetate dimeglumine was calculated pixel by pixel. This single slice technique yielded a sample of approximately 2000 pixels for dynamic investigations.

The correlation of gadopentetate dimeglumine concentration and $T_1$ relaxation rate showed excellent correlation parameters with $R_s = 0.947$, $P < 0.001$ and $r^2 = 0.955$ (Figure 2A). The in vivo relaxivity for gadopentetate dimeglumine in arterial blood was 5.53 mM$^{-1}$ s$^{-1}$. Representative concentration–time data of gadopentetate dimeglumine in tumour and in arterial blood are shown in Figure 2B. The multiexponential approach for the parametrization of the arterial input function, $C_a(t)$, provided a

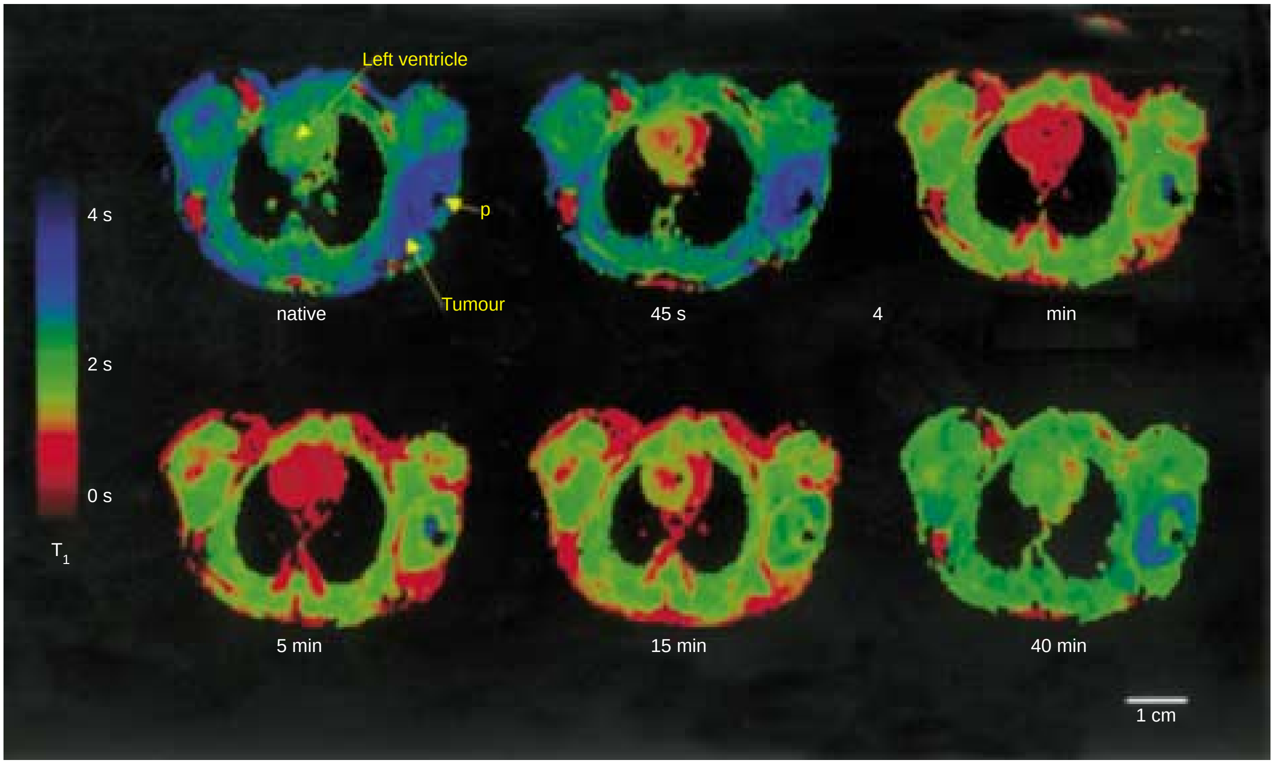

**Figure 1** Representative transaxial colour-coded T1 relaxation maps of a hamster before, during and after infusion of 0.05 mmol kg$^{-1}$ b.w. gadopentetate dimeglumine (Gd-DTPA, 0.125 ml min$^{-1}$ for 4 min). For MR imaging, an inversion recovery snapshot FLASH pulse sequences was applied (acquisition time: 2.5 s, in-plane resolution: 250 × 250 µm$^2$, slice thickness: 750 µm). From the Snapshot FLASH images, T1 relaxation time maps were calculated pixel by pixel. Regions of interest (ROIs) were defined in the centre of the cavum of the left ventricle to characterize the arterial input function, and in the tumour for analysis of tumour blood flow. A pin (p) was implanted into the tumour to facilitate orientation of tumour sections

 *© 2001 Cancer Research Campaign*

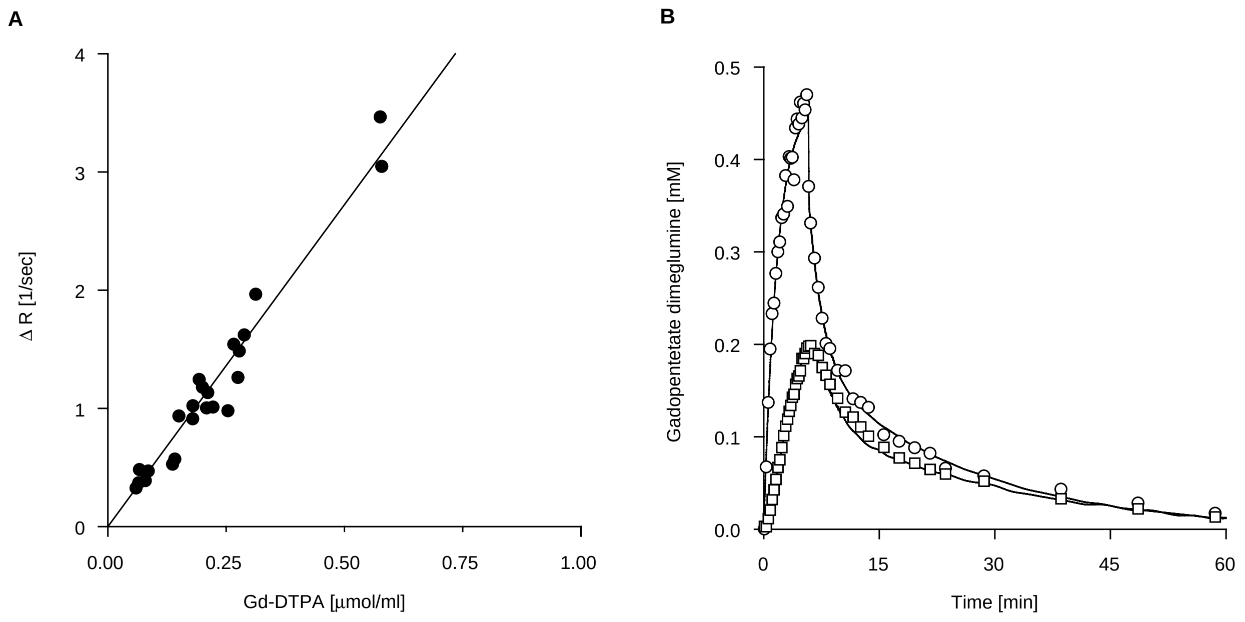

**Figure 2** (**A**) Correlation of gadopentetate dimeglumine (Gd-DTPA) concentration measured by means of inductive coupled plasma atomic spectroscopy in arterial blood samples (x-axis) and $T^1$ relaxation rates determined in the cavum of the left ventricle by means of inversion recovery snapshot FLASH pulse sequences (y-axis). Correlation parameter were as follows: $R_s = 0.947$, $P < 0.001$, $r^2 = 0.955$, y = 5.53.x. (**B**) Representative concentration time curves of gadopentetate dimeglumine in arterial blood (○) and tumour (□). Concentration of gadopentetate dimeglumine can be calculated from relaxation rates. For arterial blood, regions of interest (ROIs) were defined in the cavum of the left ventricle. The concentration time data were fitted using a nonlinear algorithm

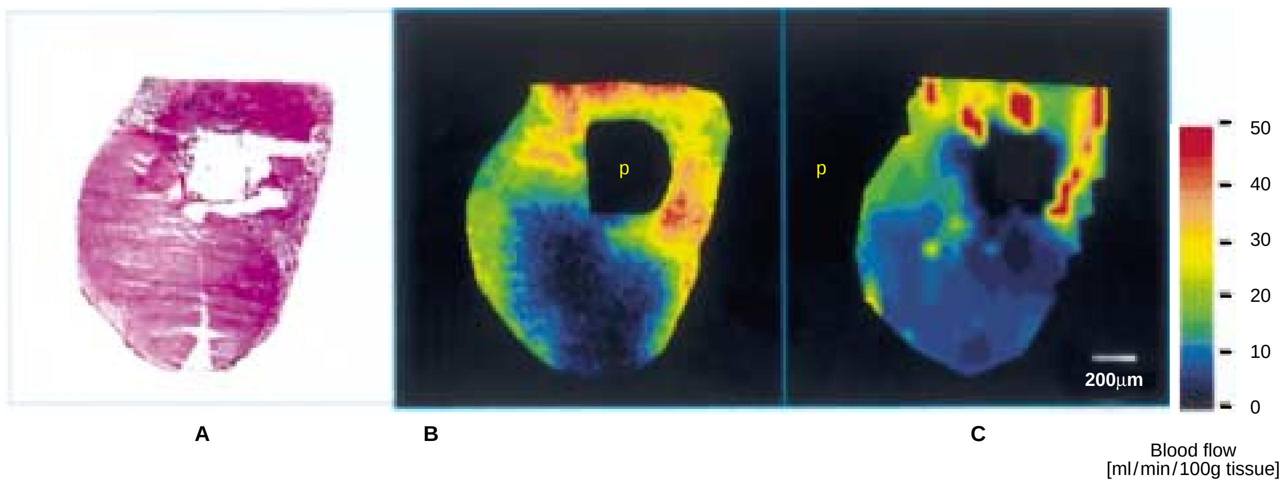

**Figure 3** Histology and corresponding colour-coded blood flow maps of an amelanotic melanoma of the Syrian golden hamster. 2 days after implantation when tumour reached palpable size a pin (p) was implanted into the tumour to facilitate orientation of tumour sections. Tumour blood flow ranged from nonperfused areas (0 ml min 100 $g^{-1}$ tumour tissue) to 'hot spots' reaching values of approximately 50 ml $min^{-1}$ 100 $g^{-1}$ tumour tissue. Iodoantipyrine autoradiography and dynamic contrast-enhanced MRI, resulted in similar images of tumour blood flow in an individual melanoma. (**A**) Cryosection stained with haematoxylin and eosin, (**B**) corresponding colour-coded autoradiograph of Iodo($^{14}$C) antipyrine representing the distribution of local tumour perfusion; (**C**) corresponding colour-coded blood flow image calculated by dynamic contrast enhanced MRI

good agreement between fitted and measured data with $r^2 > 0.9$ in all cases.

For identification of tumour tissue, histological section stained with haematoxylin and eosin were performed. Tumours could be clearly depicted compared to corresponding parameter maps obtained by MRI and autoradiography (Figure 3). Tumour blood flow was distributed heterogeneously revealing non-perfused areas with values of 0 ml $min^{-1}$ 100 $g^{-1}$ tissue and 'hot spots' reaching values of approximately 50 ml $min^{-1}$ 100 $g^{-1}$ tissue. Images of tumour blood flow from MRI and autoradiography exhibited a similar distribution in corresponding tumour sections (Figure 3). An example of the pixel-to-pixel correlation of tumour blood flow between iodoantipyrine autoradiography and dynamic contrast enhanced MRI in corresponding sections of the A-Mel-3 tumour is shown in Figure 4A. Comparison of maps resulted in a correlation coefficient of Rs = 0.851 (measuring grid: 600 × 600 $\mu m^2$, r = 0.775, $P < 0.0001$, $n = 62$). Precision and bias between these 2 measurement techniques were 5.3 and 1.9 ml $min^{-1}$ 100 $g^{-1}$ tumour tissue, respectively (Figure 4A, B).

Data of tumour blood flow demonstrated an enormous heterogeneity between tumours. Global tumour blood flow of the

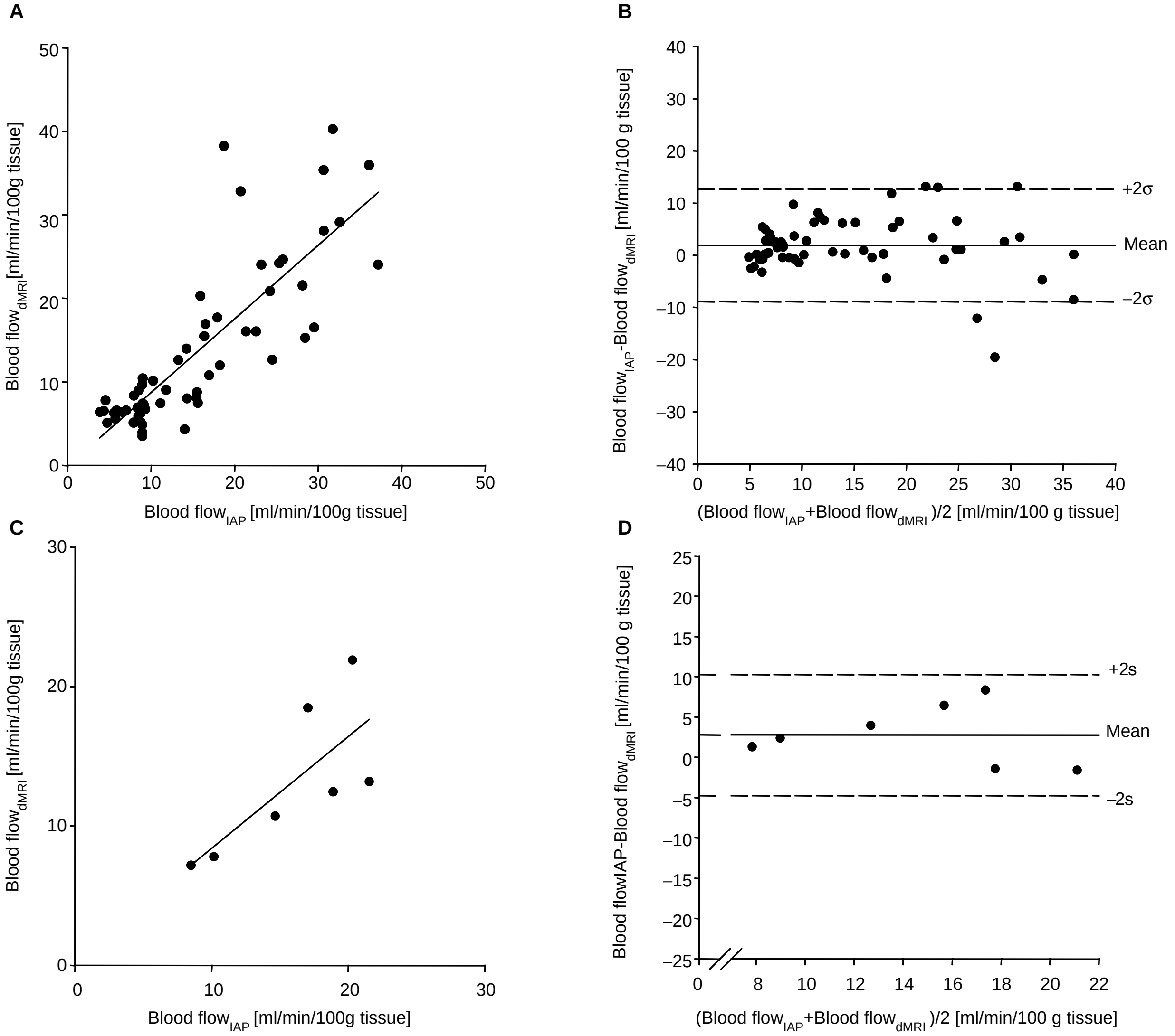

**Figure 4** (**A**) Correlation of regional tumour blood flow given in corresponding pixels of 600 × 600 μm² of perfusion maps calculated by iodoantipyrine autoradiography and dynamic contrast enhanced MRI ($R_s$ = 0.851, r = 0.775, $P$ < 0.0001, $n$ = 62; equation of the linear regression curve: y = 0.88 x – 0.11). (**B**) Illustration of agreement with respect to regional tumour blood flow between the 2 different measurement techniques by presentation of the data as Bland and Altman plot; precision: 5.3 ml min$^{-1}$ 100 g$^{-1}$ tissue, bias: 1.9 ml min$^{-1}$ 100 g$^{-1}$ tissue. (**C**) Relation between global tumour blood flow in 7 tumours measured by iodoantipyrine autoradiography and dynamic contrast enhanced MRI. Global tumour blood flow of the amelanotic melanoma was 15.9 ± 1.9 ml min$^{-1}$ 100 g$^{-1}$ tumour tissue (range: 8.5–1.5 ml min$^{-1}$ 100 g$^{-1}$ tissue) calculated by dynamic contrast enhanced MRI and 13.1 ± 2.0 ml min 100 g$^{-1}$ tissue (range: 7.8–21.9 ml min$^{-1}$ 100 g$^{-1}$ tissue) calculated by iodoantipyrine autoradiography. Correlation analyses for perfusion data between these 2 measurement techniques demonstrate good correlation ($R_s$ = 0.821, r = 0.72, $P$ = 0.014, $n$ = 7; equation of the linear regression curve: y = 0.88x + 0.37). (**D**) Illustration of agreement with respect to global tumour blood flow between the 2 measurement techniques as Bland and Altman plot; precision: 3.8 ml min$^{-1}$ 100 g$^{-1}$ tissue, bias: 2.8 ml min$^{-1}$ 100 g$^{-1}$ tissue amelanotic melanoma was 15.9 ± 1.9 ml min$^{-1}$ 100 g$^{-1}$ tumour tissue (range: 8.5–21.5 ml min$^{-1}$ 100 g$^{-1}$ tumour tissue) calculated by dynamic contrast enhanced MRI and 13.1 ± 2.0 ml min$^{-1}$ 100 g$^{-1}$ tumour tissue (range: 7.8–21.9 ml min$^{-1}$ 100 g$^{-1}$ tumour tissue) calculated by iodoantipyrine autoradiography (Figure 4C). Blood flow data of the 2 different measurement techniques revealed a good correlation for the tumours investigated (Rs = 0.821, r = 0.72, $P$ = 0.014). Precision and bias between the 2 techniques were 3.8 and 2.8 ml min$^{-1}$ 100 g$^{-1}$ tumour tissue, respectively (Figure 4D).

In addition to tumour blood flow, the extraction fraction as well as the parameters $I_b$ and $I_d$ could be quantified. The mean extraction fraction, α, over all tumours investigated, was 0.15 ± 0.03. $I_b$ and $I_d$ were 1.72 ± 0.39 min$^{-1}$ and 0.14 ± 0.02 min$^{-1}$, respectively. From these results, a mean intervascular MTT of gadopentetate dimeglumine of 0.58 min and a mean interstitial MTT of 6.67 min has been estimated.

### DISCUSSION

Dynamic MR imaging combines fast MR measurement techniques and administration of contrast agents. For analysis of uptake and washout of contrast agents in tumours by a tracer kinetic model, a linear relationship between contrast agent concentration and measured MR signal has to be ascertained. Due to the complex dependence of signal intensities upon tissue parameters, pulse sequence parameters, and contrast agent concentration, it was proposed to quantify gadopentetate dimeglumine concentration in tissue using T1 relaxation times (Diesbourg et al, 1992; Tong et al,

 *© 2001 Cancer Research Campaign*

1993; Donahue et al, 1994). For extracranial tissues, under steady-state conditions, empirical data suggest a linear relationship between longitudinal bulk T1 relaxation rate and tissue concentration of extracellular contrast agents (Strich et al, 1985; Burstein et al, 1991; Donahue et al, 1994). This corresponds with the assumption of a fast water exchange between vascular, interstitial and cellular compartments in tissue, which mediates the compartmental relaxation processes. Hence, tissue relaxation is regarded as a single bulk relaxation, which is the weighted average of the contributions of the corresponding compartments. For dynamic MR studies, however, an instantaneous bolus injection technique is widely used. Hence it has to be taken into account that extracellular contrast agents, such as gadolinium dimeglumine, are not freely diffusible. Consequently, a steady state between intravascular and interstitial contrast agent concentrations is probably not reached during the first pass of the contrast agent, and thus the assumption of a fast water exchange between vascular and extravascular spaces may be faulty (Donahue et al, 1994; Judd et al, 1995). In order to minimize this kind of error, we used a prolonged bolus injection technique, as proposed by Schmiedl et al (1991). This seems to be preferable because water exchange times between tumour compartments remain rapid enough to validate fast exchange due to balancing extravasation of contrast agents into tissue (Donahue et al, 1994).

Concentration–time data from dynamic MR imaging offers the potential to determine microcirculatory parameters if an appropriate tracer kinetic model is used, which takes into account the tracerkinetic properties of the contrast agent employed. Gadopentetate dimeglumine (Gd-DTPA), which is widely used in the clinical setting for contrast-enhanced MR imaging is characterized by a small molecular weight and its restriction to the extracellular space. In the central nervous system (CNS) gadopentetate dimeglumine behaves exactly like an intravascular tracer due to the blood–brain barrier and is, therefore, generally suitable for quantification of cerebral microvascular parameters by dynamic studies. However, outside the CNS, gadopentetate dimeglumine is rapidly extracted from the vascular into the interstitial space. The extraction fraction estimated in different organs varies between 0.2 and 0.7 (Diesbourg et al, 1992; Tong et al, 1993; Larsson et al, 1994; Daldrup et al, 1998). In our study, we found an extraction fraction of $0.15 \pm 0.03$ for the A-MEL 3 tumour tissue.

Descriptive parameters, such as the initial uptake rate, the maximum uptake, and the decay rate of the contrast agent in the tumour have been introduced to determine perfusion like parameters (Mayr et al, 1996; Hawighorst et al, 1998). These parameters might be of practical use in clinical routine because they can be simply derived from contrast agent kinetics in the tumour. However, in addition to perfusion rate these parameters also depend on a variety of other microcirculatory parameters such as extraction fraction, fraction of the intravascular and extracellular volume, as well as the arterial input function. Therefore, descriptive parameters do not reflect relative or absolute perfusion rates in the tumours (Lyng et al, 1998).

Tissue blood flow measurements can be performed by the Kety model when freely diffusible tracers are applied (Kety, 1960). For contrast agents which are not freely diffusible such as gadopentetate dimeglumine, a modification of the Kety model has been proposed as a first approach to the evaluation of myocardial (Diesbourg et al, 1992; Tong et al, 1993) and tumoural (Lyng et al, 1998) uptake curves. The major drawback of these approaches is that they do not provide blood flow values directly but rather the product of blood flow and extraction fraction (Taylor et al, 1999). As a consequence, the utility of the modified Kety method depends on the a priori knowledge of the extraction fraction which has to be determined by a separate method. Particularly in tumours, the extraction fraction is not homogeneously distributed, because it is governed by physiologic and morphologic properties of tissue and varies within the tumour dependent on grading type, vascular supply, permeability, convection and histopathologic features such as necrotic areas (Lyng et al, 1998). Due to the heterogeneity of tumours, the extraction fraction has thus to be determined on a pixel-to-pixel basis. Tracer kinetic modelling allowing simultaneous calculation of extraction fraction of gadopentetate dimeglumine and perfusion rate is highly warranted. A promising approach based on an extension of the Kety method was introduced by Larson et al (1994). Unfortunately, these algorithms are very sensitive to noise in the MR data and are hard to realize under in vivo conditions in the clinical setting.

Another approach for the estimation of microcirculatory parameters from dynamic MR studies relies on compartment analysis. As a first step in this direction, simple 2-compartment models were used (Larsson et al, 1990; Brix et al, 1991; Tofts and Kermode, 1991). However, these models did not take into account tumour blood flow. A promising approach, which overcomes this limitation and allows for the separate quantification of blood flow and permeability-surface product, was recently proposed by Brix et al (1999). A principal limitation of all compartment approaches is that they consider plasma and interstitial space as compartments, in which instantaneous mixing is assumed during the transit of tracer molecules. Although this assumption seems to be reasonable for the interstitial compartment, it is unlikely that it is valid for the transit of the arterial bolus through the vascular space. Therefore, in line with Morales and Smith (1948), a constant correction factor was introduced by Brix et al (1999) which takes into account the differences in the plasma concentration of the tracer between the arterial entry and the venous exit of the capillary mesh. The perfusion data, presented in this study for oro- and hypopharyngeal carcinomas, are in good agreement with PET measurements. However, as discussed by the authors, it is dubious to what extent the perfusion values are overestimated by their approach.

To overcome these problems a novel tracer kinetic approach (Griebel et al, 1997) was applied which is based on a model originally introduced by Meier and Zierler (1963). This approach relies on the frequency function h(t) which is the distribution of the transit times of the tracer through the tissue after an instantaneous tracer bolus. For extracellular tracers such as gadopentetate dimeglumine, which are neither intravascular, nor freely diffusible, great attention has to be directed to finding an appropriate expression for h(t). In line with Zierler (1963) h(t) can be divided into an intravascular and an extravasating fraction. The weighting between both fractions is given by the extraction fraction $\alpha$. This approach is universally applicable for freely diffusible tracers ($\alpha = 1$, Kety (1960) formula) intravascular tracers ($\alpha = 0$, formula of Meier and Zierler (1963)) as well as for intermediates ($0 < \alpha < 1$, gadopentetate dimeglumine).

In the complex in vivo investigations, performed in this study, a lot of potential biological and experimental errors can attribute to the variability of data, that is heterogeneity of tumour blood flow, dependence of tumour blood flow upon macrohaemodynamics, changes in the cardiovascular condition of the animals within the interval between MRI and reference technique, difficulties in the choice of identical ROIs within the tumour. Despite these limitations, we found a good correlation for this tracer kinetic-based contrast-enhanced

© 2001 Cancer Research Campaign

MRI technique for the quantification of tumour blood flow. The validation of the MRI technique showed good correlation with data obtained by the reference method yielding good parameters for precision and bias of the technique analysed by the Bland and Altman analysis as well as acceptable correlation parameters with $r^2 > 0.5$ by linear regression and Rs > 0.8 by Spearman range correlation.

This presented MRI technique has already been transferred to a clinical whole body 1.5 T MR scanner being a robust and practical tool for monitoring concentration time data (de Vries et al, 2000, 2001). Application of the tracer kinetic model will allow for quantitative mapping of tumour blood flow. Quantitative assessment of tumour vascularity is of utmost relevance for the efficacy of different treatment modalities. Moreover, the distinct properties of tumour vascular supply are gaining more and more attention in differential diagnosis, prognosis, therapeutic monitoring and prediction of therapeutic success. Data from perfusion-like semiquantitative MRI measurements indicate that tumour vascularity has a prognostic impact in clinical trials and can be used to individualize and monitor anticancer therapy (Mayr et al, 1996; de Vries et al, 2000, 2001). These data are very promising and exciting, and underline the potential for noninvasive quantitative measurement techniques assessing precisely tumour blood flow. Knowledge about the specific vascular microenvironment of tumours could be used for selective antivascular cancer treatment in the future.

### ACKNOWLEDGEMENTS

We are grateful to Prof Dr h.c.mult. K Messmer (Institute for Surgical Research, University of Munich, Germany), as well as to Prof Dr H Habazettl (Department of Physiology, University of Berlin, Germany), and Dr G Brix (Institute of Radiation Hygiene, Federal Office for Radiation Protection, Neuherberg, Germany) for their helpful suggestions and comments on the manuscript. Furthermore, we would like to thank Prof Dr P Schrammel (Institute for Ecological Chemistry, GSF Research Center for Environment and Health, Neuherberg, Germany) for performing the inductive coupled plasma atomic spectroscopy measurements. The study was partly sponsored by Schering AG (Germany).

### REFERENCES

Bland JM and Altman DG (1986) Statistical methods for assessing agreement between two methods of clinical measurement. *Lancet* **1**: 307–310

Brix G, Semmler W, Port R, Schad LR, Layer G and Lorenz WJ (1991) Pharmacokinetic parameters in CNS Gd-DTPA enhanced MR imaging. *J Comput Assist Tomogr* **15**: 621–628

Brix G, Bahner ML, Hoffmann U, Horvath A and Schreiber W (1999) Regional blood flow, capillary permeability, and compartment volumes. Measurement with dynamic CT-initial experience. *Radiology* **210**: 269–276

Burstein D, Taratuta E and Manning WJ (1991) Factors in myocardial perfusion imaging with ultrafast MRI and Gd-DTPA administration. *Magn Reson Med* **20**: 299–305

Daldrup HE, Shames DM, Husseini W, Wendland MF, Okuhata Y and Brasch RC (1998) Quantification of the extraction fraction for gadopentetate across breast cancer capillaries. *Magn Reson Med* **40**: 537–543

Deichmann R and Haase A (1992) Quantification of T1 Values by SNAPSHOT-FLASH NMR Imaging. *J Magn Reson* **96**: 608–612

Degani H, Gusis V, Weinstein D, Fields S and Strano S (1997) Mapping pathophysiological features of breast tumors by MRI at high spatial resolution. *Nat Med* **3**: 780–782

de Vries A, Griebel J, Kremser C, Judmaier W, Gneiting T, Debbage P, Kremser T, Pfeiffer KP, Buchberger W and Lukas P (2000) Monitoring of tumor microcirculation during fractionated radiation therapy in patients with rectal carcinoma: preliminary results and implication for therapy. *Radiology* **217**: 385–391

de Vries AF, Griebel J, Kremser C, Judmaier W, Gneiting T, Kreczy A, Ofner D, Pfeiffer KP, Brix G and Lukas P (2001) Tumor microcirculation evaluated by dynamic magnetic resonance imaging predicts therapy outcome for primary rectal carcinoma. *Cancer Res* **61**: 2513–2516

Diesbourg LD, Prato FS, Wisenberg G, Drost DJ, Marshall TP, Carroll SE and O'Neill B (1992) Quantification of myocardial blood flow and extracellular volumes using a bolus injection of Gd-DTPA: kinetic modeling in canine ischemic disease. *Magn Reson Med* **23**: 239–253

Donahue KM, Burstein D, Manning WJ and Gray ML (1994) Studies of Gd-DTPA relaxivity and proton exchange rates in tissue. *Magn Reson Med* **32**: 66–76

Fritz-Hansen T, Rostrup E, Larsson HB, Sondergaard L, Ring P and Henriksen O (1996) Measurements of the arterial concentration od Gd-DTPA using MRI: a step toward quantitative perfusion imaging. *Magn Reson Med* **36**: 225–231

Folkman J (1995) Angiogenesis in cancer, vascular, rheumatoid and other disease. *Nat Med* **1**: 27–31

Fortner JG, Mahy AG and Schrodt GR (1961) Transplantable tumors of the Syrian (Golden) hamster. Part I: tumors of the alimentary tract, endocrine glands and melanomas. *Cancer Res* **21**: 161–198

Griebel J, Mayr NA, de Vries A, Knopp MV, Gneiting T, Kremser C, Essig M, Hawighorst H, Lukas PH and Yuh WT (1997) Assessment of tumor microcirculation: a new role of dynamic contrast MR imaging. *J Magn Reson Imaging* **7**: 111–119

Griebel J, Pahernik SA, Lucht R, De Vries A, Englmeier KH, Dellian M and Brix G (2001) Perfusion and permeability: can both parameters be evaluated separately from dynamic MR data. *Proc Int Soc Mag Reson Med* **9**: 629

Goetz A (1987) Quantitative Analyse der Tumormikrozirkulation im amelanotischen Hamstermelanom A-MEL-3. *Medical thesis at the university of Munich*

Hawighorst H, Weikel W, Knapstein PG, Knopp MV, Zuna I, Schoenberg SO, Vaupel P and von Kaick G (1998) Angiogenic activity of cervical carcinoma: Assessment by functional Magnetic Resonance Imaging-based parameters and a histomorphological approach in correlation with disease outcome. *Clin Cancer Res* **4**: 2305–2311

Henderson E, Sykes J, Drost D, Weinmann HJ, Rutt BK and Lee TY (2000) Simultaneous measurement of blood flow, blood volume, and capillary permeability in mammary tumors using two different contrast agents. *J Magn Reson Imaging* **12**: 991–1003

Hoeckel M, Schlenger K, Aral B, Mitze M, Schaffer U and Vaupel P (1996) Association between tumor hypoxia and malignant progression in advanced cancer of the uterine cervix. *Cancer Res* **56**: 4509–4515

Jain RK (1998) The next frontier of molecular medicine: delivery of therapeutics. *Nat Med* **4**: 655–657

Judd RM, Atalay MK, Rottmann GA and Zerhouni EA (1995) Effects of myocardial water exchange on T1 enhancement during bolus administration of MR contrast agents. *Magn Reson Med* **33**: 215–223

Kety SS (1960) Measurement of local blood flow by the exchange of an inert, diffusible substance. *Methods Med Res* **8**: 228–236

Kuhnle GE, Dellian M, Walenta S, Mueller-Klieser W and Goetz AE (1992) Simultaneous high-resolution measurement of adenosine triphosphate levels and blood flow in the hamster amelanotic melanoma A-Mel-3. *J Natl Cancer Inst* **84**: 1642–1647

Larsson HBW, Stubgaard M, Frederiksen L, Jensen M, Henriksen O and Paulson OB (1990) Quantitation of blood-brain barrier defect by magnetic resonance imaging and Gadolinium-DTPA in patients with multiple sklerosis and brain tumors. *Magn Reson Med* **16**: 117–131

Larsson HB, Stubgaard M, Sondergaard L and Henriksen O (1994) In vivo quantification of the unidirectional influx constant for Gd-DTPA diffusion across the myocardial capillaries with MR imaging. *J Magn Reson Imaging* **4**: 433–440

Lyng H, Dahle GO, Kaalhus O, Skretting A and Rofstad EK (1998) Measurement of perfusion rate in human melanoma xenograts by contrast-enhanced magnetic resonance imaging. *Magn Reson Med* **40**: 89–98

Mayr NA, Yuh WT, Magnotta VA, Ehrhardt JC, Wheeler JA, Sorosky JI, Davis CS, Wen BC, Martin DD, Pelsang RE, Buller RE, Oberley LW, Mellenberg DE and Hussey DH (1996) Tumor perfusion studies using fast magnetic resonance imaging technique in advanced cervical cancer: a new noninvasive predictive assay. *Int J Radiat Oncol Biol Phys* **36**: 623–633

Meier P and Zierler K (1963) On the theory of the indicator-dilution method for measurement of blood flow and volume. *J Appl Physiol* **6**: 731–744

Morales MF and Smith RE (1948) On the theory of blood-tissue exchange of inert gases. VI. Validity of approximate uptake expressions. *Bull Math Biophys* **10**: 191–200

Nekolla S, Gneiting T, Syha J, Deichmann R and Haase A (1992) T1 maps by K-space reduced snapshot-FLASH MRI. *J Comput Assist Tomogr* **16**: 327–332

 *© 2001 Cancer Research Campaign*

Rempp KA, Brix G, Wenz F, Becker CR, Guckel F and Lorenz WJ (1994) Quantification of regional cerebral blood flow and volume with dynamic susceptibility contrast-enhanced MR imaging. *Radiology* **193**: 637–641

Sakurada O, Kennedy C, Jehle J, Brown JD, Carbin GL and Sokoloff L (1978) Measurement of local cerebral blood flow with iodo [14C] antipyrine. *Am J Physiol* **234**: H59–H66

Schmiedl UP, Kenney J and Maravilla KR (1991) Kinetics of pathological blood-brain-barrier permeability in an astrocytic glioma using contrast-enhanced MR. *Am J Neuro Radiol* **13**: 5–14

Strich G, Hagan PL, Gerber KH and Slutsky RA (1985) Tissue distribution and magnetic resonance spin lattice relaxation effects of Gadolinium-DTPA. *Radiology* **154**: 723–726

Taylor JS, Tofts PS, Port R, Evelhoch JL, Knopp M, Reddick WE, Runge VM and Mayr N (1999) MR imaging of tumor microcirculation: promise for the new millenium. *J Magn Reson Imaging* **10**: 903–907

Tofts PS and Kermode G (1991) Measurement of the blood-brain barrier permaebility and leackage space using dynamic MR imaging. 1. Fundamental concepts. *Magn Reson Med* **17**: 357–367

Tong CY, Prato FS, Wisenberg G, Lee TY, Carroll E, Sandler D, Wills J and Drost D (1993) Measurement of the extraction efficiency and distribution volume for Gd-DTPA in normal and diseased canine myocardium. *Magn Reson Med* **30**: 337–346

Vaupel P, Kallinowski F and Okunieff P (1989) Blood flow, oxygen and nutrient supply, and metabolic microenvironment of human tumors: a review. *Cancer Res* **49**: 6449–6465

Weidner N, Semple JP, Welch WR and Folkman J (1991) Tumor angiogenesis and metastasis-correlation in invasive breast carcinoma. *N Engl J Med* **324**: 1–8

Workman P, Twentyman P, Balmain A, Chaplin D, Double J, Embleton J, Newell D, Raymond R, Stables J, Stephens T and Wallace J (1998) United Kingdom co-ordinating committee on cancer research (UKCCCR) guidelines for the welfare of animals in experimental neoplasia (second edition). *Br J Cancer* **77**: 1–10

Zierler KL (1963) Theory of use of indicators to measure blood flow and extracellular volume and calculation of transcapillary movement of tracers. *Circ Res* **12**: 464–471

© 2001 Cancer Research Campaign 
